# Implications for OLE RNA as a natural integral membrane RNA

**DOI:** 10.1261/rna.080489.125

**Published:** 2025-08

**Authors:** Seth E. Lyon, Ronald R. Breaker

**Affiliations:** 1 Department of Molecular Biophysics and Biochemistry; 2 Department of Molecular, Cellular and Developmental Biology, Yale University, New Haven, Connecticut 06520, USA

**Keywords:** bTOR, noncoding RNA, OapA, ribonucleoprotein complex, stress resistance, transmembrane

## Abstract

Ornate, large, extremophilic (OLE) RNAs, found in many Gram-positive bacterial species, represent an unusual class of noncoding RNAs, which form a large ribonucleoprotein complex that localizes to cell membranes. Although the precise biochemical functions of OLE RNAs remain to be discovered, several lines of evidence suggest that they participate in forming particles that function as the master regulators of their bacterial hosts. Thus, OLE RNA might be a molecular relic of RNA World organisms that contributed to cellular stress responses long before the evolutionary emergence of proteins. Recent reports of partial 3D structures strongly suggest that OLE RNAs form a molecular dimer whose complex structure spans the phospholipid bilayer of membranes. The implications of these findings on the functions of OLE RNA and on the capabilities of RNA polymers more broadly are discussed.

## INTRODUCTION

OLE RNAs were initially discovered (Puerta-Fernandez et al. 2006) by using a bioinformatics search strategy (Barrick et al. 2004; Corbino et al. 2005) to reveal the existence of highly conserved RNA sequences and structures derived from the noncoding portions of bacterial genomes. OLE RNAs are not widely distributed like some other structured noncoding RNA (ncRNA) classes in bacteria, such as ribosomal RNAs (rRNAs), transfer RNAs (tRNAs), ribonuclease P RNAs (RNase P), or group I and group II self-splicing ribozymes. Rather, OLE RNAs are restricted to species of Bacillota (Firmicutes) that thrive under anaerobic or other extreme conditions (Puerta-Fernandez et al. 2006; Fernando and Breaker 2024). This phylum appears to have emerged early in the diversification of the bacterial domain of life (Coleman et al. 2021), which might mean that OLE RNAs represent an ancient type of ncRNA class even though they are more narrowly distributed than some other classes.

**FIGURE 1.**
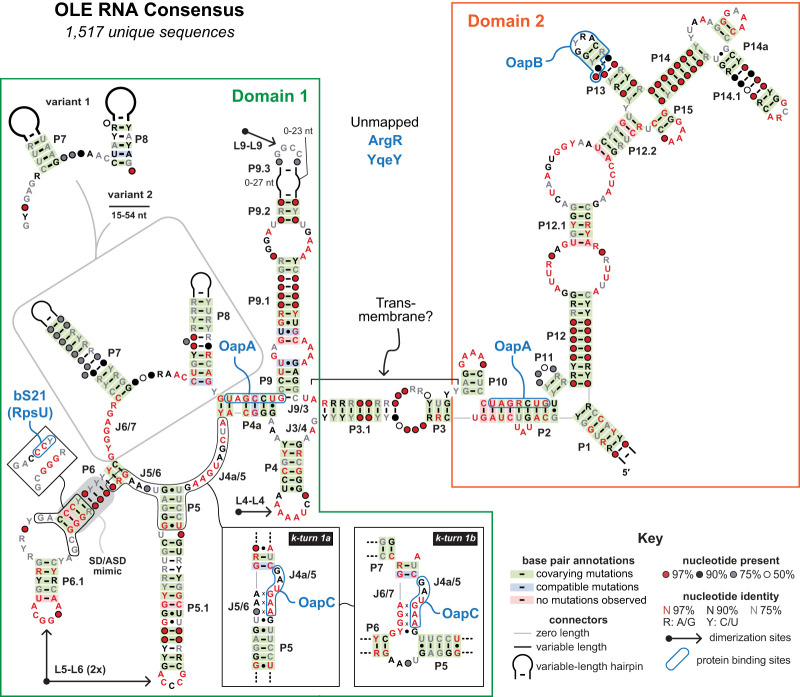
Consensus sequence and secondary structure model for OLE RNAs. Annotations include known or strong candidate protein partners (Wencker et al. 2025), alternative substructures (k-turns, *insets*) (Lyon et al. 2022; Wencker et al. 2025), RNA dimerization contact points (Kretsch et al. 2025; Wang et al. 2025), and common phylogenetic variations spanning the J6/7 through P8 region (Fernando and Breaker 2024). The secondary, tertiary, and quaternary structural features of Domain 1 have been modeled using cryo-EM data, whereas the higher-level structural features of Domain 2 remain to be determined.

On its discovery, several additional unusual characteristics (Table 1) of OLE RNAs hinted that they were likely to have one or more special functions. Most prominently, OLE RNAs are long (∼600 nt), they carry many highly conserved nucleotides (∼25%), and they form a remarkably complex secondary structure (Fig. 1). Indeed, the name of the RNA was derived from its ornate secondary structure, large size, and presence in extremophilic organisms. These features alone triggered speculation that OLE RNAs most likely function as some form of ribozyme because ncRNAs with similar sizes, structural complexities, and extents of conservation typically perform difficult or sophisticated chemical transformations (Puerta-Fernandez et al. 2006; Harris and Breaker 2018).

**TABLE 1.** Characteristics of OLE RNAs that were recognized soon after the discovery of this ncRNA class and that indicated this ncRNA would have unusual biochemical and biological functions

Characteristic	Description
Host species	OLE RNAs are often present in extremophilic, anaerobic bacteria
Length	OLE RNAs are ∼600 nt
Conservation	∼25% of the nucleotide identities are >97% conserved
2° Structure	Covariation reveals ∼25 base-paired substructures
Gene associations	*ole* gene clusters with various fundamental genes
Cellular abundance	OLE RNAs are the fifth most abundant, excluding rRNA and tRNA
RNP complex	OLE RNAs form a large ribonucleoprotein complex
Localization	OLE RNP complexes localize to the cell membrane
KO phenotypes	OLE RNA disruption causes diverse stress phenotypes

**FIGURE 2.**
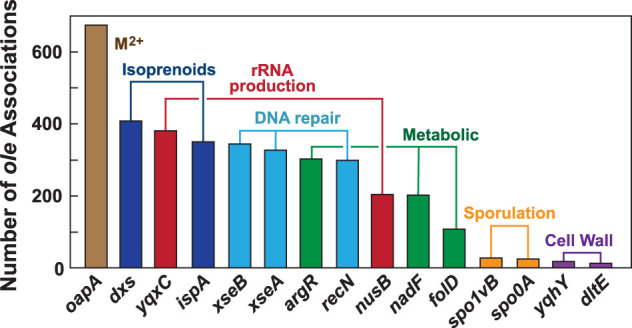
Plot of the most common genes associated with *ole* listed in order of abundance based on the analysis of 730 species producing OLE RNA (Breaker et al. 2023). Genes were counted if they reside within six genes upstream of or downstream from *ole*. The activities of the proteins encoded by these genes are linked to the broad biological processes indicated. Clustering of genes in bacteria often indicates functional connections (Okuda et al. 2007; Rocha 2008), suggesting that OLE RNA is relevant to the annotated activities of these neighboring genes.

The unusual characteristics of OLE RNAs are now known to be even more expansive (Table 1). As we noted previously (Puerta-Fernandez et al. 2006; Breaker et al. 2023), the gene for OLE RNA typically resides in a cluster with other genes that code for proteins relevant to diverse but fundamental cellular processes. Further, in the model extremophile *Halalkalibacterium halodurans* (formerly *Bacillus halodurans*), this gene cluster is transcribed as a single polycistronic RNA (Puerta-Fernandez et al. 2006). Because gene clustering in bacteria often reveals functional connections between their protein products (Okuda et al. 2007; Rocha 2008), it is likely that the functions of OLE RNA are somehow connected to this eclectic collection of gene products (Fig. 2). However, the only published link between OLE RNA and these genes is that the most common, immediate downstream neighbor of the *ole* gene is a gene now called *oapA*, which codes for a predicted dimeric membrane protein that cooperatively binds to OLE RNA (Block et al. 2011). This interaction between OLE RNA and OapA (OLE-associated protein A) is necessary for OLE RNA to localize to the membrane of its host cell. In *H. halodurans*, OapA is also necessary for the RNA to perform its known biological functions (Wallace et al. 2012; Harris et al. 2019; Lyon et al. 2024).

If OLE RNA is indeed ancient, and its complicated structure is used to carry out one or more biochemical tasks, then its biological roles must be so profound that its gene shares genomic locations with those relevant to many other fundamental processes. These processes are linked to metal ion homeostasis, isoprenoid biosynthesis, translation, DNA repair, central metabolism, sporulation, and cell wall biosynthesis (Fig. 2). And yet, enigmatically, the OLE RNP complex can be deleted from *H. halodurans* (Wallace and Breaker 2011; Wencker et al. 2024) without causing any detectable defect in growth characteristics under optimal growth conditions (Wallace et al. 2012; Harris et al. 2019; Lyon et al. 2024). This indicates that the biochemical mechanisms of OLE RNP complexes must be relevant when organisms encounter certain environmental stresses. Only then do the functions of OLE RNP complexes become as critical as the functions of the proteins whose genes are located adjacent to *ole* and *oapA*.

The OLE RNP complex from *H. halodurans* appears to be involved in sensing and/or responding to various environmental stresses, including exposure to short-chain alcohols or cold (Wallace et al. 2012), high Mg^2+^ concentrations (Harris et al. 2019), and suboptimal carbon/energy sources (Lyon et al. 2024), among others (Breaker Laboratory, unpubl.). Based on these and other clues, our research team proposed (Breaker et al. 2023) that OLE RNP complexes are functionally like mTOR complexes of eukaryotes, which enable cells to respond to numerous different stresses (Sabatini 2017; Liu and Sabatini 2020). We have called the RNA-containing devices “bTOR” (bacterial tasks OLE regulates) in recognition of these functional similarities to mTOR, although there does not appear to be any homology between components of the eukaryotic and bacterial complexes.

We speculate that OLE RNAs fulfill their biochemical tasks through interactions formed with OapA and likely many additional proteins. Like OapA, two additional proteins called OapB (Harris et al. 2018; Widner et al. 2020; Yang et al. 2021) and OapC (Lyon et al. 2022) bind OLE RNA and are essential for OLE RNA function in *H. halodurans*. These two proteins could be simple RNA folding chaperones, although their precise roles in the OLE RNP complex remain unclear. Furthermore, OLE RNA “pulldowns” (Lyon et al. 2022) revealed that the RNA interacts with several other proteins in *H. halodurans.* We recently determined that one of these interacting proteins, ribosomal protein bS21, forms a biologically relevant contact with OLE (Wencker et al. 2025), providing a physical link between the OLE RNP complex and a protein involved in the process of translation. The experimental validation of additional protein partners such as ArgR (Breaker Laboratory, unpubl.), or other strong candidates such as ribosomal proteins uS7 and uS11, PnpA, and others, would implicate OLE RNP complex functions in even more fundamental biological processes.

**FIGURE 3.**
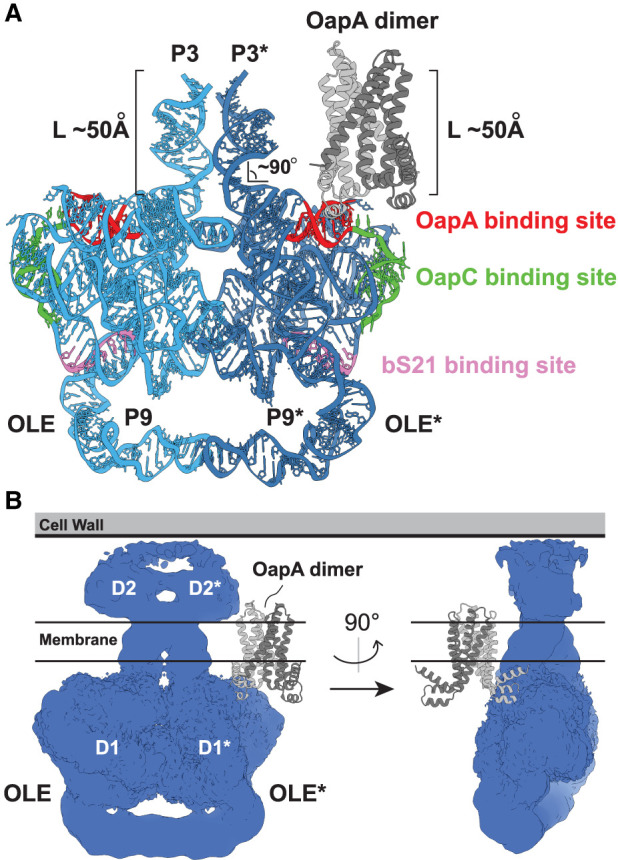
Cryo-EM models of OLE RNA. (*A*) High-resolution model for domain 1 of the OLE RNA from *Clostridium botulinum* based on cryo-EM data reported recently (Wang et al. 2025; PDB 9LCR). Similar results are observed for the RNA from *C. acetobutylicum* (Kretsch et al. 2025). The locations of the binding sites for OapA (Block et al. 2011), OapC (Lyon et al. 2022), and bS21 (Wencker et al. 2025), as determined by biochemical assays, are also depicted. A high-resolution model for domain 2 is not available. (*B*) Electron density map observed for a complete *C. botulinum* OLE RNA dimer, revealing that electron density for domain 2, although at low resolution, is best accommodated on the opposite side of the cell membrane (depicted here as bounded by the *horizontal* lines). The OapA dimer structure from *C. botulinum* (CLB_RS09010) was predicted using AlphaFold3 (Abramson et al. 2024). The N-terminal signal sequence of OapA was not included in the structure prediction.

There are now more surprises emerging regarding OLE RNAs. Two research teams have reported partial cryo-EM structures of OLE RNAs, providing several new insights regarding this unusual ncRNA class. First, two OLE RNAs form an RNA dimer via the formation of several highly conserved tertiary contacts. Second, about two-thirds of each RNA can be modeled at high resolution and, when dimerized via multiple intermolecular contacts, the RNAs form an unusually flattened structure (Fig. 3). However, the remainder of the RNA near the 5′ and 3′ ends forms a separate domain that cannot yet be structurally resolved at high resolution. Third, and most strikingly, the models proposed for OLE RNA dimers suggest that the elongate P3 region of each RNA spans the lipid bilayer, which would mean that the two distinct domains of the OLE RNA dimer reside on opposite sides of the cell membrane. The implications of this remarkable structural model are discussed herein.

## EVIDENCE THAT OLE RNA ADOPTS A TRANSMEMBRANE CONFIGURATION

The genomic colocalization of *ole* and *oapA* genes provided an early indication that OLE RNAs were likely to be bound by OapA and localized to cell membranes (Puerta-Fernandez et al. 2006). OapA is predicted to form four transmembrane helices and has a conserved cluster of positively charged amino acids on its cytoplasmic face (Puerta-Fernandez et al. 2006; Harris et al. 2018) that were speculated to be involved in OLE RNA binding. RNP complex formation was confirmed (Block et al. 2011) by biochemical assays demonstrating that *H. halodurans* OapA binds to OLE RNA from this same species. The data from these initial protein-binding assays suggested that OapA is cooperatively bound by OLE RNA and forms at least a 2:1 protein-to-RNA complex. Furthermore, fluorescence imaging revealed that OapA is necessary for OLE RNAs to localize to the membranes of surrogate *Escherichia coli* cells.

Until now, the simple models of OLE RNP complexes were conservatively depicting an OapA dimer embedded in a phospholipid bilayer and complexed with a single OLE RNA (Harris et al. 2018). Based on conventional thinking, the RNA was assumed to reside on the cytoplasmic side of the membrane. After each new protein partner (OapB, OapC, bS21) was identified, these were also depicted near their mapped binding sites (Widner et al. 2020; Yang et al. 2021; Lyon et al. 2022; Wencker et al. 2025). Dimerization of OLE RNAs had at least been briefly considered previously. For example, experiments with an OLE RNA fragment containing the OapB-binding site exhibit a propensity for dimerization (Widner et al. 2020). We and others had also observed OLE RNA multimerization or aggregation in various experiments, although these observations had remained unpublished.

Recent cryo-EM structures of OLE RNA (Kretsch et al. 2025; Wang et al. 2025) reveal that these early models and assumptions were far too conservative. The new partial structural models are based on the analysis of OLE RNAs from two different species (*Clostridium acetobutylicum* and *C. botulinum*), which reveal similar features. For example, the left portion of each OLE RNA (base-paired substructures P3 through P9) forms an intricate, globular structure (here called “domain 1”) with four dimerization locations (Figs. 1, 3). One of these centers involves A-stacking and sheared A•A pairing between the conserved A-rich loops of the P4 stems of both RNAs. The second and third centers are formed by duplicate kissing-loop interactions between the conserved loop nucleotides of P5.1 in one OLE RNA and the complementary loop nucleotides of P6.1 in the other OLE RNA. The fourth center is formed by a kissing-loop interaction between the conserved, symmetrical loop sequences of P9 stems. This latter interaction uses the unusually long P9 stem substructures to form an arch-like architecture that is held apart from the main, tightly packed globular structure. Conserved nucleotides are distributed at several locations in P9, suggesting that these nucleotides and the unusual arch-like structure of the dimer perform some additional unknown function(s). Neither P7 nor P8 serves as dimerization contact points, and this might explain why some natural OLE RNAs lack these substructures but presumably can still perform their key biological functions (Fernando and Breaker 2024).

Conspicuously, the globular domain 1 structure exhibits a notably flat surface on the side near the P4 dimerization center, as if the structure has evolved to avoid a clash with another large object (specifically, the cell membrane) (Fig. 3). In addition, both cryo-EM models indicate that the P3 stems of the dimer project out from the flat plane formed by domain 1, creating an angle with P4 of ∼90°. Given that one of the two predicted binding sites for OapA is in a region originally called P4a, this transmembrane protein is predicted to bind immediately adjacent to P3, and in a parallel orientation to this protruding stem. Surprisingly, the only reasonable model to accommodate the cryo-EM structural model with OapA bound is for both P3 stems of the RNA dimer to penetrate through the lipid bilayer of the membrane (Fig. 3).

Ignoring for now the obvious challenge of positioning negatively charged phosphate of RNA within the hydrophobic interior of a lipid bilayer (see further discussion below), there is another important question to address. Is the P3 stem of OLE RNA long enough to span a bacterial membrane? The glycerol-to-glycerol distance of a typical phospholipid bilayer is ∼35 Å (Lee 2003). Similarly, the entire width of a membrane (distance between phosphates) composed of phosphatidylcholine with palmitate acyl groups (C16) is ∼37 Å (Lewis and Engelman 1983). Given that there are ∼2.5 Å rise per RNA base pair in an A-form helix (Griffith 1978), ∼14 bp would be needed to span the hydrophobic region of a typical phospholipid bilayer. The combination of stems P3 and P3.1 typically sums to 12 bp, with additional spanning distance coming from the small internal bulge between these two stems and the joining regions called J3/4 and J9/3 (Fig. 1). Based on these estimates, OLE RNAs project a helical structure that indeed appears to have sufficient length needed to extend entirely across the bacterial membrane.

Furthermore, the cryo-EM models for the OLE RNA dimer reveal that both P3 stem structures project ∼50 Å from domain 1 (Fig. 3A). This means that domain 2, encompassing substructures P1, P2, and P10 through P15 (Fig. 1), must either be (i) on the opposite side of the membrane from domain 1, (ii) make another pass across the membrane, (iii) reside on the cytoplasmic side with domain 1 because the OapA protein is modeled in the wrong location, or (iv) be removed by RNA processing events. At this time, there is no evidence for the latter three scenarios. Although existing cryo-EM data do not permit domain 2 to be modeled at high resolution, the data do indicate that this region also dimerizes to form a flattened globular structure in the space immediately at the protruding ends of the P3 stems (Fig. 3B). Thus, the structural data, which show a severe size constriction in OLE RNA near the OapA-binding sites, best fit with scenario (i). This constriction would limit the number of negatively charged RNA nucleotides exposed to the hydrophobic interior of a phospholipid bilayer.

## IS MEMBRANE INSERTION OF RNA BIOCHEMICALLY POSSIBLE?

To date, there are no natural RNAs that are known to remain embedded in cell membranes. Of course, there are various mechanisms by which RNAs exit the nucleus of eukaryotes to perform their functions in the cytoplasm (Köhler and Hurt 2007). In addition, RNAs can exit cells via membranous compartments (O'Brien et al. 2020) or by other mechanisms (Jose 2015; Shugarts Devanapally et al. 2025) and be taken up by other cells. Some recently discovered RNAs are even bound to membrane exteriors (Flynn et al. 2021; Chai et al. 2023), further broadening the interplay between RNA and cell barriers. However, when known, these processes all involve specialized protein factors or pores that permit RNAs to pass beyond phospholipid bilayers.

Associations between RNAs and synthetic phospholipid membranes without protein partners have been demonstrated previously. Directed evolution methods were used to create populations of small RNAs that partition with phosphatidylcholine synthetic liposomes (Khvorova et al. 1999) and can be observed colocalizing with the phospholipid bilayer (Janas and Yarus 2003). Some members of the pool evolved to form trimeric RNA complexes (Vlassov et al. 2001) that structurally collaborate to bind vesicles and alter their permeability. These results indicate that certain RNA molecules can localize to phospholipid bilayers without the assistance of proteins, small molecules, or chemical modifications to their polynucleotide chain. The fact that these RNA interactions erode the integrity of the phospholipid bilayers reveals that they can disturb the normal barrier functions of membranes. However, it is not known whether the mechanism of disruption involves RNAs penetrating the membrane.

RNA residency within membranes should be strongly disfavored unless supportive interactions with the charged or hydrophilic portions of phospholipids are balanced against the disadvantages caused by placing negative charges of RNA phosphodiester linkages adjacent to hydrophobic fatty acid tails of the membrane's interior. Perhaps the severe energetic penalty could be mitigated if a chemical barrier existed between the putative transmembrane domain of OLE RNA and the hydrophobic interior of the lipid membrane. It seems plausible that such a shield could be composed of aliphatic molecules such as polyamines or membrane protein(s), such as OapA. However, if OapA proteins are limited to a 2:1 ratio with OLE RNA and a 4:2 ratio with OLE RNA dimers, then there does not appear to be sufficient protein to fully shield the exposed surface area of the two membrane-penetrating P3 stems (Fig. 3A). Because the initial experimental data on OapA binding to OLE RNA revealed that the interactions were highly cooperative, we concluded that the stoichiometry between protein and RNA was “at least 2:1” (Block et al. 2011). Thus, the stoichiometry might be even higher in favor of protein to RNA (see further discussion below). It remains possible, however, that one or more additional membrane-associated proteins participate in enabling OLE RNA to stably penetrate the membrane.

## IMPLICATIONS FOR A TRANSMEMBRANE OLE RNA CONFIGURATION

Beyond the biochemical challenges of inserting and maintaining a large ncRNA in a bacterial membrane, there are many other aspects of OLE RNA structure and function that will need to be addressed by reason or by experiment. Several of the most prominent issues are outlined below.

### The structure of OLE RNA domain 2

The fact that OLE RNAs from two different species cannot be fully modeled with existing cryo-EM data suggests that other interactions necessary for domain 2 folding are missing. We know that at least one missing partner is OapB (Harris et al. 2018), which binds portions of stem P12.2 and the stem and loop of P13 (Fig. 1; Widner et al. 2020; Yang et al. 2021). However, there are many highly conserved segments in domain 2 that are not involved in this simple RNA–protein interaction. Some of these nucleotides could serve as RNA dimerization sites, as is observed for domain 1. Regardless, there could be additional interactions with protein factors, or with biochemical components of the peptidoglycan cell wall of Gram-positive species—which should be within reach beyond the periplasmic space of even a highly folded dimer of domain 2 (Fig. 3B).

### The OapA–OLE RNA complex

Partial binding sites for OapA proteins were mapped to two palindromic sequences present in or near P2 and P4a of OLE RNA (Block et al. 2011), and these sites flank the possible transmembrane helical domain formed by P3 and P3.1. The palindromic binding sites on each OLE RNA in the dimer are now expected to be on opposite sides of a phospholipid bilayer. This creates a problem for RNP assembly because an individual OapA dimer cannot be modeled to simultaneously interact with their predicted OLE RNA–binding sites on a single RNA molecule. If the true protein-to-RNA ratio is 4:2 and each OapA protein binds one palindromic RNA sequence, then two OapA proteins need to be oriented with their OapA-binding sites facing inward while the remaining two need to be facing outward.

If OapA proteins dimerize in the same orientation, and if these dimers function as divalent metal ion transporters, then the OLE RNP complex might help create an antiparallel ion flow system. Under some cellular conditions, ions might be permitted to flow into the cell via one orientation of the OapA dimer, and under other conditions, the same ions are ejected from the cell via the other dimer orientation. Alternatively, OapA proteins could dimerize in an antiparallel fashion. Two such protein dimers likewise could engage all four binding sites on the OLE dimer. Computational methods (AlphaFold) produce OapA dimers in either orientation (Fernando and Breaker 2024; data not shown), and therefore we cannot easily rule out either of the models proposed above.

**FIGURE 4.**
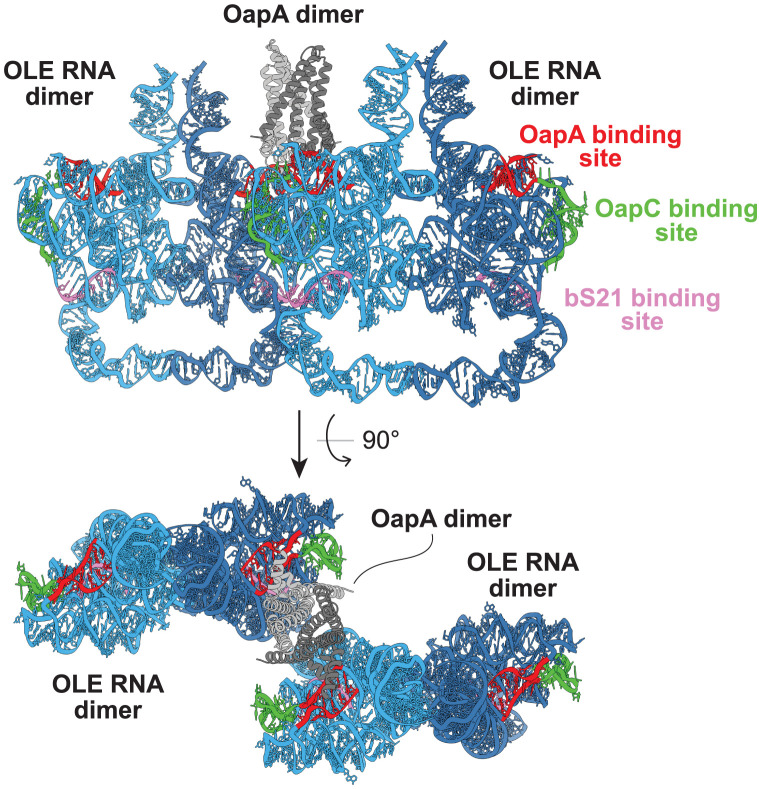
Model depicting the possible bridging of two OLE dimer particles via an OapA dimer. This arrangement involves a parallel-arranged OapA dimer wherein both polypeptides engage a predicted docking site at P4a. Repeating these interactions would yield an extended sheet of partially overlapping OLE RNA dimers. Individual components of this hypothetical model are as described in Figure 3.

Another major consideration is that a single OapA dimer cannot physically span the distance between the OapA-binding sites in the two P4a regions of a single OLE RNA dimer. This poses an interesting possibility—that each OapA dimer binds to two OLE RNA dimers. The flattened architecture formed by domain 1 of OLE RNA dimers means that two RNP complexes could approach sufficiently close that an OapA dimer can span the distance between two P4a regions in *separate* OLE RNA dimers (Fig. 4). This stacked arrangement could be expanded so that OLE RNP complexes could be aggregating to form sheets or patches of interacting particles in the membrane. Unfortunately, the number of possible protein–RNA arrangements that could be imagined is very large, and the room for speculation is mostly unconstrained by existing data sets.

### The changing shapes of OLE RNAs

The recent cryo-EM structural models indicate that the domain 1 portions of OLE RNA dimers form an intricate and compact structure, with the exception of the P9 arch (Fig. 3). Importantly, both phylogenetic and biochemical data indicate that OLE RNA molecules adopt multiple different structures, consistent with the hypothesis that RNA shape changes can be triggered by cellular conditions or protein binding events. For example, the binding of either OapC (Lyon et al. 2022) or bS21 (Wencker et al. 2025) causes OLE RNA to alter its initial conformation involving simple P5 and P6 stems into one of two potential k-turn structures (Fig. 1, see insets). The cryo-EM structures also reveal 14 electron densities presumably corresponding to bound Mg^2+^ ions (Wang et al. 2025). Because OLE RNA knockouts are sensitive to excessive Mg^2+^ concentrations, it is possible that intracellular divalent metal ion concentrations could stabilize the dimeric, bridged form of OLE RNA. These shape changes could be harnessed by the RNA to influence the binding affinities of protein partners, the structures and functions of these partners, or other biochemical interactions of relevance. If true, then researchers can expect many forms of OLE RNP complexes to exist in cells, wherein each is distinguished by its constellation of protein partners and RNA structural features.

### Linking stresses of multiple compartments and barriers

If OLE RNA indeed extends from the cell interior, through the membrane, and reaches all the way to make physical contact with the cell wall, then its functions might involve the evaluation of the status of each of the barriers it touches and the compartments it passes through. About two-thirds of the RNA (domain 1) on the cytoplasmic side could provide plenty of exposed surface area and structural interplay to accommodate protein or even small chemical binding sites (aptamers). Membrane proteins (in addition to OapA) or the lipid bilayer itself could interact with the portions of OLE that reside in or near the membrane space. The remainder of OLE RNA (domain 2) seems certain to form a complex structure due to its notable conservation of sequence, secondary structure features, and its known interaction with OapB (Yang et al. 2021). Biochemical substituents of the periplasmic space and components of the cell wall might selectively interact with this portion of OLE RNA to assist in the folding and function of domain 2. Indeed, interactions between OLE RNP partners in one location might trigger RNA shape changes that propagate signaling and actions in another location.

Currently, there is little direct evidence for such a complicated function of the OLE RNP complex. However, there are a few notable observations that make this hypothesis worth considering. For example, bS21 binding to OLE RNA domain 1 has been linked to the ability of OLE RNA to respond to ethanol and cold stresses (Wencker et al. 2025). Perhaps the interplay between bS21 binding to OLE RNA and its similar interaction with ribosomes (also present in the cytoplasm) help explain its stress response effects. The interaction of OLE RNA with OapA, a putative divalent metal ion transporter (Breaker et al. 2023; Fernando and Breaker 2024), might be necessary for transport of a specific ion type across the membrane. Finally, among the other high-ranking genes associated with OLE RNA are *yqhY* and *dltE* (Fig. 2). We have previously noted (Breaker et al. 2023) that these two genes code for proteins that are relevant to cell wall stress responses and to cell wall production, respectively. Changes in cell wall structure or composition might be evaluated via direct contact with portions of domain 2.

### OLE RNP complexes versus MpfA-family proteins

There is growing evidence supporting the initial speculation (Harris et al. 2019) that members of the MpfA family of proteins (metal ion transporters) are distally related to OapA proteins and that they exhibit functions similar to OLE RNP complexes. Genes for OLE RNAs anticorrelate with genes for MpfA proteins as revealed by an extensive genome analysis (Fernando and Breaker 2024). Bacterial strains carrying genetic knockouts of MfpA-family proteins exhibit phenotypes (Armitano et al. 2016; Breaker Laboratory, unpubl.) that are like those observed for OLE knockout strains (Harris et al. 2019). Finally, some phenotypes caused by disrupting OLE RNP function can be rescued by certain MpfA-family proteins, and vice versa (Breaker Laboratory, unpubl.).

Given the findings above, MpfA-family proteins appear to be all-protein versions of perhaps more ancient OLE RNP devices (Fernando and Breaker 2024). If true, then it might be possible to speculate on the roles of OLE RNA by comparing it to MpfA protein domains. The similarities between OapA and MpfA-family proteins reside in their transmembrane region, or the CNNM domain of MpfA. However, MpfA-family proteins also often carry additional protein domains: tandem CBS domains (Kemp 2004; Baykov et al. 2011; Sureka et al. 2014) and a HlyC/CorC domain (Bateman et al. 2018). We have previously proposed that OLE RNAs might perform some of the same functions as the MpfA domains missing from OapA (Harris et al. 2018; Fernando and Breaker 2024). Perhaps these putative functions can now be localized to domain 1 of OLE RNA. However, MpfA-family proteins are missing an extension at their N-terminus that would serve as the functional equivalent of OLE RNA domain 2. Regardless, learning more about one of these systems is likely to provide insight into the functions of the other.

## MAJOR QUESTIONS

As more details of OLE RNP complexes are uncovered, several major questions become apparent. Here, we briefly discuss three prominent questions that we believe merit special attention.

### Precisely how does OLE RNA penetrate the membrane?

As discussed above, RNAs are often moved across membrane barriers with the aid of specialized protein factors. If OLE RNP complexes must reside in cell membranes to carry out their biological functions, then genetic screens already conducted (Harris et al. 2018; Harris et al. 2019; Lyon et al. 2024) should have revealed such factors—that is unless these are essential for cell survival. The only known proteins essential for OLE RNP function in *H. halodurans* are OapA, OapB, and OapC. OapA is a putative divalent metal ion transporter (Harris et al. 2019; Breaker et al. 2023; Fernando and Breaker 2024) with a very simple architecture that seems unlikely to be an active catalyst for inserting a large ncRNA into membranes. OapB is a ∼100 amino acid polypeptide that binds domain 2 of OLE RNA. If this protein remains bound to OLE RNA in its final, active state, it would be located in the periplasmic space. In contrast, OapC is ∼80 amino acids long and binds to a domain 1 location placing it on the cytoplasmic side of the membrane. Alone, these small proteins also seem unlikely to be the active agents of OLE RNA insertion into the membrane.

In surrogate *E. coli* cells, expression of OLE RNA alone does not result in membrane localization (Block et al. 2011). However, the coexpression of OapA with OLE RNA causes OLE RNA to localize to the cell periphery, presumably to the membrane. If this localization involves membrane insertion, then only OLE RNA and OapA are sufficient for membrane insertion, or there fortuitously is a system in *E. coli* that can achieve this same task. This brings up the possibility that OapA and OLE RNA might represent the only two components necessary to achieve membrane insertion of RNA.

### Why does OLE need to be an integral membrane RNA?

At this time, there is no evidence that portions of OLE RNAs exist that function independently of the larger OLE RNP complex. This suggests that it is essential for OLE RNAs to span from the peptidoglycan cell wall to the cytoplasmic space of host cells using contiguous RNA molecules. Our original speculation was that OLE RNA might serve as a ribozyme (Puerta-Fernandez et al. 2006), only because nearly all large, well-conserved, and ancient RNAs are known to catalyze chemical transformations (Harris and Breaker 2018). However, the demands placed on an RNA that must serve as an integral membrane RNA could explain its remarkable size, structure, and conservation.

As discussed above, one possible reason for a transmembrane architecture is that OLE RNAs might be important for communicating physical or biochemical changes between the cell wall, the membrane, and the compartments in which portions of the RNAs reside. Another intriguing possibility is that the RNA directly functions as a transporter RNA that selectively recognizes its target and facilitates its movement across the membrane. Indeed, could the OLE RNA dimer function as a divalent metal cation transporter in a manner equivalent to MpfA proteins (Chen et al. 2021)? Again, given the diversity of biological processes linked to the OLE RNP complex, there are too many possibilities for RNA functions coupled with too few constraints that could accurately guide such speculations. Therefore, a more comprehensive understanding of the structures and functions of the OLE RNP complex are required to address this question.

### What is the full scope of OLE RNA biological functions?

Bioinformatics, biochemistry, and genetics analyses have yielded some links between OLE RNP complexes and their biological purposes. Sufficient progress has been made to support an initial theory for its purpose—that these unusual devices function as master regulators of cells to coordinate physiological responses to various stress conditions (Breaker et al. 2023). The current understanding of these functions is likely to be far from complete. For example, there still is no unifying functional theme that spans the growth defect phenotypes that occur under several diverse stresses. Furthermore, only the *oapA* and *argR* genes from the *ole* gene cluster (Fig. 2) have been biochemically and genetically linked to the OLE RNP complex—so what about all the others? Genetic selections indicate that more proteins are linked to OLE RNP function (Harris et al. 2018, 2019; Lyon et al. 2024), as do RNA “pull-down” results (Lyon et al. 2022). If such connections are confirmed, what can these additional clues indicate about the roles of OLE RNA?

With the insights gained from the cryo-EM structures, questions about biological functions might be guided by considering how an integral membrane RNA can help achieve the cell's objectives. Already we know that OLE RNAs can adopt multiple, biologically relevant shapes (Lyon et al. 2022; Wencker et al. 2025). Therefore, the problem of establishing the full scope of biological functions is complicated by the fact that OLE RNP complexes are likely to be dynamic with regard to structure, protein partners, and perhaps even the formation of larger molecular assemblies (Fig. 4). The dynamics of OLE RNP particles might be observable inside cells by new technologies such as cryo-ET imaging, which would also help reveal the complete collection of protein partners that are relevant to its biological and biochemical functions.

## CONCLUDING REMARKS

Currently, there are too many uncertainties regarding the structural models for OLE RNA to speculate with confidence on the full scope of features and functions of OLE RNP complexes. However, existing evidence strongly suggests that OLE RNAs form a very large and complicated RNP complex that projects through cell membranes. If correct, OLE RNAs would represent the first known integral membrane RNAs.

Given that OLE RNAs interact with multiple protein components, change shapes when these interactions occur, and appear to form structures that penetrate cell membranes, the extensive sequence and structure conservation they exhibit is at least partly justified. However, there are plenty of structural features and conserved regions within OLE RNA dimers that have yet to be assigned a biochemical role. Therefore, we believe that there are many additional opportunities to discover new biological functions, biochemical contacts, and perhaps even binding sites for small molecule effectors or active sites for ribozymes. Gaining a complete understanding of this remarkable and likely ancient RNA device will require the application of numerous strategies and techniques—with surprising findings certain to result. These efforts are warranted if OLE RNP complexes are indeed the master regulators of its host cells.

It is fascinating to consider the origin of OLE RNA and what its roles might have been early in evolution. Although OLE RNA could be an evolutionary invention that happened after the emergence of proteins, it seems more likely that such a complex and highly structured RNA might have performed similar functions during the RNA World. If modern cells greatly benefit from a master regulator of cell physiology such as seen with mTOR and bTOR devices, then advanced RNA World organisms might also have gained an advantage by having a large ncRNA like OLE projecting through whatever these cells used as their membranes.
